# Alarming Rate of Substance Use in Motor Vehicle Collisions at an Appalachian Trauma Center

**DOI:** 10.7759/cureus.11863

**Published:** 2020-12-03

**Authors:** Rebecca Proctor, Melissa P Taylor, Megan Quinn, Bracken Burns

**Affiliations:** 1 Surgery, Quillen College of Medicine, East Tennessee State University, Johnson City, USA; 2 Epidemiology and Public Health, College of Public Health, East Tennessee State University, Johnson City, USA

**Keywords:** controlled substances, motor vehicle collision, substance abuse, appalachia

## Abstract

Prescription drug use is a growing public health concern and studies show it is a contributing risk to motor vehicle collisions. The Appalachian region is also known to have an ever-increasing number of patients on controlled substances. This retrospective study of patients from the years 2011-2015 on controlled substances presenting to an Appalachian Level 1 trauma center after a motor vehicle or motorcycle collision was analyzed in order to determine the rate of opioid use among victims of motor vehicle collisions in the system, as well as evaluate for any differences in resource utilization between these patients and patients not using controlled substances.

A total of 2,570 patients were included in the study. Seven-hundred sixty-eight (29.9%) individuals were found to be on a controlled substance. There was a similar mortality rate in both groups (2.8% vs 3.6%). There was no significant difference in hospital length of stay (LOS), intensive care unit (ICU) LOS, ventilator days, or injury severity score. Statistically significant findings include the type of crash (motor vehicle crash vs motorcycle crash) (p=0.003) and position in the vehicle (driver vs passenger) (p<0.001). Motor vehicle crashes and driver position were significantly associated with the presence of a controlled substance.

## Introduction

The most recent published data from the National Highway Traffic Safety Administration (NHTSA) indicates a national increase of 6% traffic fatalities per year or 11.59 fatalities per 100,000 people. In the state of Tennessee, this increase of fatalities was found to be even higher than the national average, with a rise of 8% contributing to 15.65 fatalities per 100,000. Of the factors identified to be related to this increase in fatalities, driving too fast (17.8%) and being found to be under the influence of alcohol, drugs, or medication (10.8%) were the most commonly cited. Seven percent to 15% of drivers involved in traffic fatalities tested positive for illegal drugs or prescription medications [1].

Rudisill et al. in a meta-analysis identified buprenorphine, codeine derivatives, tramadol, methadone, benzodiazepines, carisoprodol, levocetirizine, zolpidem, and zopiclone as having an increased risk of motor vehicle collisions (MVC). Twelve of the 15 medications cited are known to inhibit the ability to maintain a position in a lane, miss instructions or directions, impair the ability to maintain speed, delay reaction time, and misconceive gaps between cars [2]. Furthermore, Guohua and Chihuri found that opioid use significantly increased the rate of fatal crash involvement, independent from alcohol use [3].

Prescription and non-prescription drug abuse, particularly opioids and benzodiazepines, is problematic nationwide. In particular, the incidence of substance use disorder and related complications is higher in the Appalachian region as compared to the national average. The Appalachian region is broadly defined as following the spine of the Appalachian Mountains from southern New York to northern Mississippi. In Tennessee, the opioid overdose rate was 19.3 deaths per 100,000 persons in 2017, higher than the national rate of 14.6 deaths per 100,000 persons [4]. Tennessee is among the highest in the nation for prescribing opioids, with 94.4 opioid prescriptions per 100 persons; yet, the rate nationwide is only 58.7 per 100 persons [5]. The rate in Tennessee has been slowly decreasing but is still well above the national average. Our trauma center lies in Southern Appalachia in Northeast Tennessee. The mostly rural population consists of patients from the greater tri-cities area that includes patients from Johnson City, Kingsport Tennessee, and Bristol Tennessee/Virginia.

Most data regarding substance use and motor vehicle collisions are obtained from events in which there is a fatality. The NHTSA Fatality Accident Reporting System (FARS) conducts data in which there was a fatality from a motor vehicle accident within 30 days and does not evaluate national drug use trends due to inconsistent drug testing laws between states [1]. NHTSA also performs a National Roadside Survey of randomly selected drivers in the contiguous 48 states. The most recent 2013-2014 report found 22% of drivers on potentially impairing drugs [6]. While there is a significant body of literature on opioid-related motor vehicle collisions that involve fatalities, there is a paucity of literature available on in-hospital outcomes and resource utilization by these patients. This is likely multifactorial. A literature review reveals that there is an inconsistency between states regarding routine toxicology screening of patients arriving at the emergency department after trauma and that the stigma associated with drug abuse leads to unreliable self-reporting among patients [7]. In addition, many patients will receive opioids in the field prior to transfer to the trauma center, which would interfere with the results of routine drug testing. Without a reliable means of assessing opioid use among patients, it becomes more difficult to study outcomes data in a trauma population. In an attempt to further evaluate in-hospital outcomes and utilization of resources within a level I trauma center in the Appalachian region of Tennessee, a retrospective study of patients on controlled substances presenting after a motor vehicle or motorcycle collision was performed.

The Office of National Drug Control Policy in conjunction with the Department of Transportation established the National Drug Control Strategy in an effort to reduce illicit drug use and its consequences in the United States. It aims to reduce drugged driving by 10% between 2011 and 2015. Within the state of Tennessee, there has been only a 0.5% decrease [8]. Combating drugged driving is a complex task with many factors to consider, including dosage required for impairment, available toxicology testing equipment, drug impairment recognition by law enforcement, and so on. Prescription opioids are becoming more commonly used and most commonly obtained by individuals through friends or relatives (70%) [5,8]. Recent legislation aimed at decreasing the number of prescriptions made by physicians for opioids will hopefully limit the number of prescription pills in circulation (SB 2257, 2018) [9]. In addition, as our study showed that drivers were more likely to be on a controlled substance, extensive advisement on the part of the physician should be made on driving avoidance while on controlled substances.

## Materials and methods

Description of the data

This study was approved by the East Tennessee State University institutional review board (IRB). The primary aim of this study was to assess if substance use was related to higher utilization of resources. Data were obtained from the National Trauma Registry of the American College of Surgeons (NTRACS) for all motor vehicle crashes from 2011-2015. Patient age, gender, arrival date, position in the vehicle (driver, passenger, unknown), county of the crash, crash type (motorcycle, motor vehicle), controlled substance use (yes or no), injury severity score, hospital length of stay (LOS), disposition, mortality, and, if applicable, and intensive care unit LOS and ventilator days were collected for each patient from archival medical records. A controlled substance was defined as any substance on the Federal Drug Enforcement Administration controlled substance schedule. Controlled substance use was categorized as “yes” if the patient self-reported controlled substance use (prescribed or not), the medical record indicated the patient was on controlled substances, or the toxicology screening was positive for controlled substance use prior to the administration of opioid medications. Patients in which no laboratory information with positive findings to be classified as “yes” or denied controlled substance use were classified as “no.” Patients with no known information on substance use who did not fit the criteria of a “yes” or a “no” were classified as “unknown.” Pre-hospital trip tickets and trauma flowsheets were used to determine if a patient was given opioids on the scene or in the emergency department (ED) prior to analysis. Primary endpoints measured were injury severity score (ISS), hospital LOS, ICU LOS, and ventilator days.

Statistical methods

Data were analyzed using IBM SPSS Statistics version 22. Descriptive analyses were completed to describe the study population. Frequencies and percentages were reported. A chi-square analysis was completed to determine variables that were significantly associated with controlled substance use. Means and standard deviations for hospital length of stay, ICU LOS, ventilator days, and injury severity scores for those on controlled substances and those not on controlled substances were computed. Simple and multiple logistic regression analyses were completed to determine what factors were associated with increased odds of controlled substances being present in the patient while controlling for gender. Odds ratios and corresponding confidence intervals were reported.

## Results

There were a total of 2,570 motor vehicle crash or motorcycle crash patients from 2011-2015 in the data (Table 1). Of those, 768 (29.9%) individuals were found to be on a controlled substance. The majority of crashes occurred in a motor vehicle (82.0%) as compared to a motorcycle (18.0%), were the driver of the vehicle (74.7%) as compared to the passenger (23.2%), and were male (60.7%) as compared to female (39.3%). The type of crash (p=0.003) and position in the vehicle (p<0.001) were significantly associated with the presence of a controlled substance.

**Table 1:** Description of vehicle crash data, 2011-2015

N=2570	
Variable	N%
Gender	
Females	1011 (39.3%)
Males	1559 (60.7%)
Crash Type	
Motorcycle	462 (18.0%)
Motor Vehicle	2108 (82.0%)
Mortality	
Alive	2477 (96.4%)
Dead	93 (3.6%)
Position in Vehicle	
Passenger	596 (23.2%)
Driver	1919 (74.7%)
Unknown	55 (2.1%)
Controlled Substance	
Yes	768 (29.9%)
No	1716 (66.8%)
Unknown	86 (3.3%)

The average injury severity score (ISS) was 11.91, with a standard deviation of 10.69 and a range from 1- 75. Hospital length of stay (LOS) averaged at 5.25 days, with a standard deviation of 10.86 and a range from one to 402 days. Table 2 describes the average outcomes of ISS, hospital LOS, ICU LOS, and ventilator days for those with controlled substances present and those without controlled substances present. Slight differences are observed in these outcomes, however, no statistical significance was identified.

**Table 2:** Descriptive statistics on outcomes by the presence of a controlled substance

	Controlled Substance (N=768)		No Controlled Substance (N=1716)	
	Mean (SD)	Range	Mean (SD)	Range
Hospital Length of Stay (LOS)	5.27 (7.66)	1-67	5.3 (12.22)	1-402
Intensive Care Unit LOS	1.70 (4.60)	0-49	1.96 (5.65)	1-112
Ventilator Days	1.25 (5.00)	0-66	1.47 (6.30)	1-112
Injury Severity Score	11.47 (10.68)	1-75	12.11 (10.65)	1-75

Thirty-three percent (33%) of all drivers were found to be positive for controlled substance use. Multiple logistic regression analyses (Table 3) showed that drivers were 76% more likely to be on a controlled substance as compared to passengers (OR 1.76, CI 1.41-2.20). Motorcyclists were 31% less likely to be under the influence of controlled substances as compared to operators of motor vehicles (OR 0.69, CI 0.54-0.87).

**Table 3:** Factors associated with controlled substance use; multiple logistic regression *Sample included only those with known information on controlled substances and position in the vehicle **Statistically significant, p<0.01

	(N=2433)*	
	OR	Confidence Interval
Gender		
Females (reference)		
Males	0.85	0.71-1.03
Crash Type**		
Motorcycle	0.69	0.54-0.88
Motor Vehicle (reference)		
Position in Vehicle**		
Passenger (reference)		
Driver	1.76	1.41-2.20

## Discussion

With growing public health concerns regarding both controlled substance use and increasing motor vehicle fatalities, we aimed to evaluate the rate of controlled substance use in patients presenting to an Appalachian level 1 trauma center after a motor vehicle collision as well as their in-hospital outcomes. While there has been extensive research on the use of controlled substances as a risk factor for motor vehicle collision and their use in fatal traffic accidents, our study appears to be one of the first to evaluate outcomes in all patients with controlled substance use that survive to presentation. Most patients positive for controlled substance use in this study were involved in motor vehicles as compared to motorcycles, were found to be male more often than female, and were more likely to be the driver of the vehicle than the passenger. There were no statistical differences in outcomes measured, including ISS, LOS, ICU LOS, ventilator days, and mortality. It was found that 29.9% of patients were using some form of controlled substance, as determined by self-reporting, pharmacy records, or toxicology screens.

One of the limitations of this study involves the reliability of information regarding the use of opioid medications. Again, most of the literature on vehicle crashes involving opioid use centers around crashes with fatalities. This is mainly due to the fact that fatalities are consistently evaluated for substances on toxicology screens while those being treated or admitted to the hospital setting are often not tested with a toxicology screen. Emergency departments often utilize patient-obtained histories as the source for substance use, rather than widespread blood or urine testing, due to the inaccuracies of such tests, the confounding variables of medications administered in route or in the emergency department, as well as the legal and reimbursement ramifications of testing patients who are altered without their consent [10]. Tennessee abides by Uniform Accident and Sickness Policy Provision Law (UPPL), which allows insurance companies to deny payment for drug and alcohol-related injuries [10]. Additionally, officers may request testing if they have reasonable grounds to be concerned that a driver is under the influence (TN code §55-10-406). While this epidemiological data is available from the state of Tennessee, it is not helpful in the evaluation of an individual trauma center population. Furthermore, the institutional policy at our institution is to perform drug testing only when law enforcement requests it (the results of which are often unavailable) or when there is clinical concern for withdrawal, overdose, or adverse drug reactions. Therefore, it is difficult to know if the data set evaluated fully captured all patients involved in motor vehicle collisions that were on controlled substances.

A recent study by Chihuri found that only six states in the nation perform drug testing on at least 80% of fatally injured drivers. In this study, 23.9% of drivers tested positive for non-alcohol drugs [11]. This was slightly lower than our findings that 29.9% of all patients evaluated within this Appalachian trauma system were on a controlled substance. This is consistent with regional data for opioid dependency, as previously discussed. The Chihuri study also found that drivers under the influence of controlled substances were more likely to be male (77.8% of males vs 74.4% of females), which is consistent with our data (60.7% of males vs 39.3% of females).

In addition, as our study showed that drivers were more likely to be on a controlled substance, extensive advisement on the part of the physician should be made on driving avoidance while on controlled substances.

## Conclusions

While there were no differences in in-hospital outcomes for patients on controlled substances versus those not on controlled substances in this study, there was a large number of drivers under the influence of a controlled substance presenting to our Appalachian trauma center, even with the suspicion of under-reporting of drug use. Further investigation into this epidemic of opioid use and abuse should be done. Future efforts should explore the use of medical resources, including evaluating what percentage of patients are discharged to home or to skilled nursing facilities or rehabilitation centers, what percentage of patients have health insurance versus those that are uninsured and, therefore, present a larger burden on the healthcare system, as well as whether these patients are out of work for a protracted amount of time or not. These economic burdens are part of the equation when looking at the cost of the opioid epidemic. Whenever possible, patients should be referred to substance abuse and addiction counseling resources, and more of these need to be available to patients in the heaviest hit regions. In addition, efforts should be undertaken to evaluate the proportion and outcomes of trauma patients after motor vehicle collision per drug classification, with the goal of reducing drug- and alcohol-related motor vehicle collisions and fatalities.
